# Assessment of a pro-healing stent in an animal model of early neoatherosclerosis

**DOI:** 10.1038/s41598-020-64940-2

**Published:** 2020-05-19

**Authors:** Philipp Nicol, Christoph Lutter, Anna Bulin, Maria Isabel Castellanos, Tobias Lenz, Petra Hoppmann, Anna Lena Lahmann, Roisin Colleran, Kristina Euller, Kristin Steigerwald, Stefanie Neubauer, Florian Rechenmacher, Beatrice Stefanie Ludwig, Michael Weinmüller, Garry Kerch, Liang Guo, Qi Cheng, Eduardo Acampado, Tobias Koppara, Horst Kessler, Michael Joner

**Affiliations:** 1 Klinik für Herz- und Kreislauferkrankungen, Deutsches Herzzentrum München, Technische Universität München, München, Germany; 2 Klinik und Poliklinik für Innere Medizin I, Klinikum rechts der Isar, Technische Universität München, München, Germany; 3 DZHK (German Centre for Cardiovascular Research), partner site Munich Heart Alliance, München, Germany; 4 Institute for Advanced Study, Department of Chemistry and Center for Integrated Protein Science, Technische Universität München, München, Germany; 5 Riga Technical University, Riga, Latvia; 6 CVPath Institute Inc., Maryland, USA; 7 Department of Orthopedics, University Medical Center, Rostock, Germany; 8 Klink für Nuklearmedizin, Klinikum rechts der Isar, Technische Universität München, München, Germany

**Keywords:** Interventional cardiology, Cardiovascular diseases

## Abstract

**Background:** Neoatherosclerosis represents an accelerated manifestation of atherosclerosis in nascent neointima after stenting, associated with adverse events. We investigated whether improved reendothelialization using RGD-coated stents results in diminished vascular permeability and reduced foam cell formation compared to standard DES in atherosclerotic rabbits. **Methods and Results:** Neointimal foam cell formation was induced in rabbits (n = 7). Enhanced endothelial integrity in RGD-coated stents resulted in decreased vascular permeability relative to DES, which was further confirmed by SEM and TEM. Cell culture experiments examined the effect of everolimus on endothelial integrity. Increasing concentrations of everolimus resulted in a dose-dependent decrease of endothelial cell junctions and foam cell transformation of monocytes, confirming the relevance of endothelial integrity in preventing permeability of LDL. **Conclusion:** Incomplete endothelial integrity was confirmed as a key factor of neointimal foam cell formation following stent implantation. Pro-healing stent coatings may facilitate reendothelialization and reduce the risk of neoatherosclerosis.

## Introduction

Stent implantation is an important therapeutic tool to treat obstructive atherosclerotic coronary lesions^1,2^. However, bare metal stent implantation was associated with an inacceptable incidence of restenosis due to neointimal hyperplasia^3^. This led to the development of drug-eluting stents (DES), devices that potently inhibit neointimal hyperplasia. However, the last 10–15 years has shown us that the undoubted success of this technology in preventing restenosis is delivered at the collateral cost of delayed arterial healing, a pathophysiological entity which underlies a spectrum of adverse clinical events including late stent thrombosis, vasomotor dysfunction of the coronary arteries and neoatherosclerosis^4–6^.

Neoatherosclerosis has been described as a unique form of atherosclerosis, representing a further and accelerated manifestation of atherosclerotic disease in nascent neointimal tissue in the aftermath of stent implantation^6^. A previous autopsy study demonstrated that in-stent neoatherosclerotic changes occur earlier after DES implantation compared with bare metal stenting, although the underlying reasons remain unclear^4^. In search of causative factors, it was recently suggested that incomplete integrity of the endothelial monolayer following DES implantation may result in increased permeability for lipids and inflammatory cells. Consequently, leakiness of the endothelial monolayer after DES implantation could be regarded as important precondition for neoatherosclerotic formation. We have previously shown that polymer-free immobilization of cyclic RGD peptide on self-expanding Nitinol stents results in integrin-dependent endothelial coverage of strut surfaces^7^. Consequently, we aimed to investigate whether this pro-healing integrin coated stent accelerates re-endothelialization, resulting in diminished vascular permeability and decreased endothelial leakiness in comparison to standard drug-eluting stents in atherosclerotic rabbits. Additionally, we performed adjunct cell cultures studies to understand how the presence of anti-proliferative substances is leading to delayed vascular healing.

## Materials and Methods

Please see also supplemental for further details regarding materials and methods.

### Overall study flow

Supplemental Fig. 1 gives an overview of the different animal studies used in this work: study 1 investigated neointimal foam cell formation in bare metal stents (n = 14) using optical coherence tomography (OCT) and histopathology (n = 7 rabbits). Study 2 compared drug-eluting stents (EES, n = 5) with customized integrin αvβ3 ligand coated stents (ICS, n = 5) regarding re-endotheliasation and endothelial leakiness by using scanning and transmission electron as well as confocal microscopy (SEM/TEM and CM, n = 5 rabbits). Animal procedures were approved by the by the Upper Bavarian government (“Regierung von Oberbayern”) as well as the Institutional Animal Care and Use Committee of the Medstar Research Institute and conformed to the position of the American Heart Association on use of animals in research and the Guide for the Care and Use of Laboratory Animals published by the U.S. National Institutes of Health.

### Study 1 - rabbit model of neointimal foam cell formation (n = 7)

Male New Zealand White Rabbits (3.0 to 4.0 kg, Hazeltov, Denver, PA, n = 7 rabbits), 3 to 4 months of age, were fed an atherogenic diet (1% cholesterol and 6% peanut oil, F4366-CHL, Bio-Serv Inc) for 5 weeks to induce hyperlipidemia. Animals were then switched to reduced cholesterol (containing 0.025% cholesterol) after 5 weeks (day 35) for a total of 13 weeks. Rabbits were monitored daily for adequate and sufficient dietary intake and loss of appetite. Balloon injury followed by stent implantation of both iliac arteries (BMS, n = 14, ProKinetic Energy, 3.0 × 15 mm, Biotronik, Bülach, Switzerland, strut thickness 80 µm) was performed at 1 week. Repeat denudation of the stented arterial segments was performed 8 weeks after stent implantation (day 63) using a 3 F Fogarty catheter. Intravascular imaging with optical coherence tomography (OCT) was performed within the stented segments after 13 weeks (day 91). Euthanasia was induced directly afterwards by an overdose of pentobarbital, while rabbits were in deep anesthesia. Stented vessels were submitted for methyl methacrylate embedding (MMA) and then for standard histopathology as described below. Blood was collected for measurement of serum cholesterol at 0, 7, 35, 63, and 91 days. Two animals died due to cholesterol-induced liver failure and were excluded from final analysis.

### Study 2 - proof of principle study investigating endothelialisation between EES and ICS (n = 5)

Everolimus eluting stents (EES, n = 5, Xience Prime, 3.0 × 15 mm, Abbott Vascular, CA, strut thickness 81 µm) and customized integrin αvβ3 ligand coated stents (ICS, n = 5, 3.0 × 15 mm, strut thickness 80 µm) were randomly allocated to iliac arteries of male hypercholesterolemic New Zealand White Rabbits (n = 5) after 7 days for a duration of 12 weeks, in accordance to study 1. As coating ligand, the cyclic RGD (Arg-Gly-Asp) peptide, c(RGDfK), a highly selective ligand for the αvβ3 integrin was functionalized by the incorporation of a spacer-linker unit at the lysine residue. After plasma treatment of the stents, they were immersion coated with the functionalized peptide, c(RGDfK) Ahx Ahx 1 (4 isothiocyanatophenyl)thioureidyl, facilitating anchorage via the isothiocyanate group to the amine groups of chitosan forming a thiourea link. Prior to that, ProKinetic Energy BMS were spray-coated with chitosan-polylactide copolymer (also see supplemental material, section „Coating of stents with αvβ3 integrin ligand”). Endothelial permeability was assessed by FITC-dextran (250/500 kDa) injected 1 hour prior to euthanasia at day 91 (after 13 weeks). After euthanasia and tissue harvest, stented vessel (n = 10; 5 EES and 5 ICS) were bisected longitudinally and analysed using confocal microscopy (CM), followed by scanning and transmission electron microscopy (SEM/TEM).

### Light and immunofluorescence microscopy

MMA embedded sections were cut at 5μm thickness and stained with hematoxylin-eosin (H&E) and Movat Pentachrome. Immunoflourescent staining of endothelial cells was performed by labelling against CD31 (Dako Corp., Via Real - USA). Samples were initially incubated in 0.1% Triton X for 20 minutes and rinsed with PBS. The stent half was then subsequently exposed overnight at 4 °C to anti-CD31 (Dako Corp., Via Real – USA; dilution 1:20). The antibody reaction was visualized with an Alexa Fluor 555 donkey anti-mouse secondary antibody (Life Technologies, Carlsbad, CA dilution 1:150). DAPI (Life Technologies, Carlsbad - USA) was used as the nuclear counter stain. Selected cross-sections from rabbit iliac arteries were also stained with antibodies against RAM-11 (Dako Corp., Via Real – USA) to identify macrophages and foam cells.

### Histopathological assessment of stented arteries

Stented vessels from study 1 were examined for neointimal foam cell infiltration as well as additional features of atherosclerotic plaque formation in rabbits. From each vessel, three histological sections (proximal, middle and distal part) were investigated. Histological sections were screened for the presence of neointimal foamy macrophages and assigned an ordinal score from 0 to 4 based on the presence of foam cells along the vascular circumference (0= absence of foam cells, 1= less than 25% of circumference occupied by foam cells, 2 = 25–50% of circumference, 3 = 50–75% of circumference, 4 = greater than 75% of circumference occupied by foam cells) and the depth of foam cell accumulation relative to the endoluminal surface (0=absence of foam cells, 1= less than 25% of foam cells penetrating into the deeper neointimal layer, 2 = 25–50% of foam cells, 3 = 50–75% of foam cells, 4 = greater than 75% of foam cells penetrating into the deeper neointimal layer). Strut-based inflammation was graded as previously described^8^. Presence of distinct neointimal features such as microcalcification, hemorrhage, cholesterol clefts and neovascularization was assessed nominally and expressed as percentage of all scored quadrants^4,9^. For morphometry, the lumen and stent area and areas within the external and internal elastic lamina (EEL/IEL), were measured by computerized morphometry. The neointimal area was calculated as: (stent area minus lumen area) and the percent stenosis was calculated as: [1-(lumen area/stent area)] * 100. The neointimal thickness at and between each stent wire site was measured, and the mean neointimal thickness for each arterial segment was calculated.

### Confocal microscopy (CM), scanning electron and transmission electron microscopy (SEM/TEM)

Confocal microscopy (CM) and scanning electron microscopy (SEM) was performed as previously described^7,10,11^. Please see supplemental materials for additional information.

For transmission electron microscopy (TEM), fixed stented segments were cut at 1 μm and stained with 1% Toludine Blue in 1% sodium borate to select regions of interest. The selected portion of the stented artery was then trimmed to an area of interest and ~100 nm thick sections were cut using an ultra-microtome (Leica EM UC6rt) and placed on formvar coated 200 mesh copper grids. Grids (3 per segment; 9 per stent) were stained with 4% uranyl acetate in 35% methanol and Reynolds lead citrate. Grids were viewed on a Hitachi H-7650 Transmission Electron Microscope. Ultrastructural examination was performed at 15.000x and 40.000x magnification to qualitatively assess the morphology of endothelial cells at the luminal surface, the presence of inflammatory cells, the number of SMC layers and the presence of endothelial cell-cell contacts.

### Optical coherence tomography and data analysis

Intravascular imaging with optical coherence tomography (OCT) was performed in all BMS in study 1 in both the stented iliac arteries prior to termination using commercially available OCT imaging systems (also see supplemental materials). All arterial segments were examined with the observer blinded to the treatment group. Stents were evaluated by morphometry and computerized planimetry was performed on all stented sections similar to previously described methods^12^ (also see supplemental materials).

### Cell culture experiments

All cell culture experiments described below were performed three times. Human umbilical vein endothelial cells (Cell Applications, USA) were thawed by standard technique and grown in endothelial cell growth medium (Promo Cell, Germany) with endothelial cell growth supplement containing 5% fetal calf serum, 4 μL/mL heparin, 10 ng/mL epidermal growth factor, 1 μg/mL hydrocortisone, 50 μg/mL gentamycin sulfate, and 50 ng/mL amphotericin B, at humidified 5% CO_2_ atmosphere. Human coronary artery endothelial cells (Cell Applications, USA) were also thawed by standard technique and grown in endothelial cell growth medium (Promo Cell, Germany). In all experiments, HUVECs or HCAECs at passage 2–5 were used. HUVECs were generally used to establish assay owing to ease of access and availability of this cell line, while actual experiments were performed with HCAECs to increase translatability of our results. HUVECs or HCAECs were seeded at a concentration of 200,000/mL on semipermeable membranes (Corning Transwell polycarbonate membrane inserts, Sigma-Aldrich, USA) and incubated at 37 °C and 5% CO_2_. Monocytes (Adult Human Elutriated Monocytes, Advanced Biotechnologies Inc., Eldersburg, USA; 20,000/ml) were thawed in a 37 °C water bath and transferred to a 15 mL conical tube containing DMEM (+high glucose, +4 mM L-glutamine; Sigma-Aldrich, USA) supplemented with 10% heat inactivated human AB serum (Sigma-Aldrich, USA) and 20% heat inactivated fetal bovine serum (Sigma-Aldrich, USA). After centrifugation for 10 minutes (150 G) monocytes were seeded in^2^ 12.5 ml^2^ cell culture flasks in the presence of 500 ng/ml M-CSF to allow differentiation into macrophages (Sigma-Aldrich, USA). The cells were then maintained at 37 °C with 5% CO_2_ for 5–8 days before they were transferred in 12-well culture plates at 10^5^ cells per well using standard detachment technique.

### *In vitro* permeability assay (transwell model)

*In vitro* surfaces were coated with a commercially available linear peptide RGD (PEPTITE-2000, Advanced BioMatrix Inc., Carlsbad, USA), which is known to promote cell attachment^13^. Negative control coatings employing a non-specific peptide sequence were used to confirm the integrin-dependent anchorage of cells. Endothelial integrity was assessed by culturing HUVECs or HCAECs on semipermeable membranes (permeability assay) with a pore size of 0.4 µm (Corning Transwell polycarbonate membrane inserts, Sigma-Aldrich, USA). Confluent HUVECs and HCAECs were seeded on Transwell inserts and cultured with 500 µl medium in the upper chamber and 1500 µl medium in the lower chamber. After cells were arranged in a confluent monolayer, cell culture medium was replaced by medium supplemented with everolimus (ERL) in different concentrations (10 nM, 100 nM, 1 µM, 10 µM and 100 µM) for 24 h. Following this treatment, 10 µg/ml of fluorescently labelled LDL (Alexa Fluor 488 AcLDL, Life Technologies, USA) was added to fresh medium (upper chamber) supplying the HUVEC or HCAEC monolayer after rinsing with PBS. Finally, LDL concentrations in the upper and the lower chamber were measured after six hours of incubation at 37 °C and 5% CO_2_ using a spectrophometer calibrated by standard curve with reference samples in fluorescent light mode. For confocal microscopy, cells were then fixed and stained within the intact transwell chambers before membranes were carefully cut out of the inserts and transferred to glass slides.

### Immunofluorescent staining

To assess the attachment of HUVECs/HCAECs on RGD coated surfaces a nuclear staining using DAPI (4′,6-Diamidino-2-Phenylindole, Dihydrochloride, Life Technologies, USA) was applied. For visualization of endothelial cell shape and junctions, VE-Cadherin staining was performed. After fixation (1:1 acetone-methanol), Triton X (1% in PBS) was used to permeabilize cells. Following a 10 min blocking step with 1% bovine serum albumin (BSA, diluted in PBS), a goat anti-human VE-cadherin primary antibody (Santa Cruz Biotechnology, Inc.) was used at a dilution of 1:200 in PBS with 1% BSA and incubated overnight at 2–8 °C. A polyclonal donkey anti-goat secondary antibody (Alexa 555, Life Technologies, USA, Dilution 1:150) was then applied to visualize cell shape and junctions. Phalloidin coupled to Alexa 488 (Life Technologies, USA, Dilution 1:30) incubated at 1:30 dilution for 30 minutes was used to stain the actin-cytoskeleton of cells. Automated quantification of cell density was done by using a customized software algorithm (ImageJ 1.5, NIH, USA).

### Lipid loading of macrophages

To further investigate passage of lipoproteins through leaky endothelial cell junctions and their potential to transform macrophages into foam cells, co-cultures with human monocytes were performed. Transwell inserts with endothelial cells pre-incubated with everolimus (10 nM, 100 nM, 1 µM, 10 µM and 100 µM) for 24 hours were transferred to 12-well plates in which differentiated macrophages (see section “Cell culture eexperiments”) had been cultured for 5–8 days. Endothelial cells were carefully rinsed with PBS and supplied with fresh media before transferring them to monocyte cultures to avoid everolimus toxicity.

Prior to lipid loading, cell culture media was switched to serum free RPMI (including 1% Nutridoma-SP (Sigma-Aldrich, USA) and 1% penicillin-streptomycin). Endothelial cells were subsequently incubated with medium containing Alexa Fluor 488 AcLDL at 10 µg/ml concentration for 24 hours to allow trans-endothelial passage of AcLDL particles. Both supernatants (upper and lower chamber) were collected and the cells were fixed in 10% formalin for oil-red-O staining. A total of four independent experiments with different monocyte donors were performed.

### Oil red O staining of lipid in macrophages

Deposition of lipids was determined by Oil red O (ORO) staining. Monocytes were rinsed with PBS and fixed in 10% formalin for 10 minutes before adding 100% propylene glycol for 10 minutes. Propylene glycol was then removed and cells were stained in filtered ORO solution for 1.5 hours at room temperature. Finally, cells were differentiated in 85% propylene glycol for 5 minutes and rinsed in distilled water before being photographed. Automated quantification of lipid particles was done by using a customized software algorithm (ImageJ 1.5, NIH, USA).

### Statistical analyses

For study 1, descriptive statistics were applied to reveal median with interquartile range of ordinal data derived from histopathological scoring assessment. Nominal data are presented as relative frequency (percentage). Continuous data are presented as median with interquartile range. For study 2, continuous data were checked for normality of distribution using Wilk-Shapiro test and expressed as means with standard deviation in case of normal distribution and median with interquartile range in case of non-parametric distribution. Wilcoxon Kruskal-Wallis rank sums test was used to calculate the significance of differences between medians of non-parametric data, while ANOVA was used for group comparison of parametric data.

For cell culture studies, descriptive statistics were applied to plot individual data points (LDL concentration in μg/ml) as a function of everolimus concentration; means of all experiments (red dot) are shown along with individual data points (black dots).

## Results

### Histopathological features in atherosclerotic rabbits

**Figure 1:**
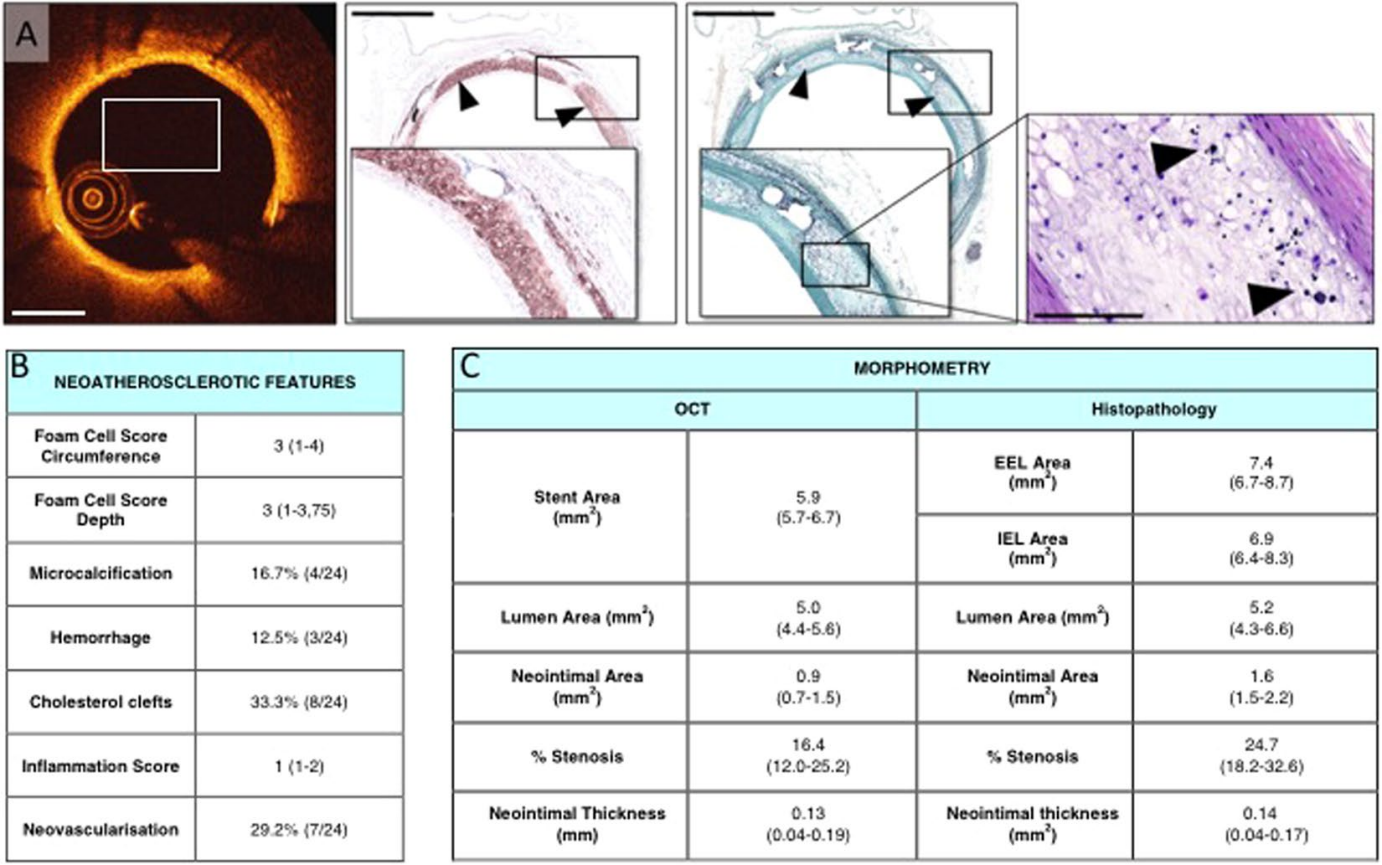
(**A**) ProKinetic Energy BMS in a rabbit iliac artery 12 weeks after implantation, assessed by histopathology and OCT. OCT shows surface with almost circumferential high backscattering intensity and attenuation. Corresponding histological cross section (Movat Pentachrome staining) shows circumferential foamy macrophage accumulation in a moderately thickened neointimal tissue (arrowheads indicate foamy macrophages; scale bar = 1000 µm). High-magnified image of Hematoxylin Eosin staining shows microcalcifications between foamy macrophages (scale bar = 100 µm). (**B**) Neointimal characteristics from study 1 and **(C)** morphometric analysis derived from OCT and histopathology (n = 5 rabbits, 24 quadrants scored in total).

Circumferential and depth of foam cell accumulation was significant, with foam cells being mostly observed within the peri-strut regions and the neointima above stent struts. Assessment of neointimal foam cells showed a mean score of 3 for both circumferential as well as depth infiltration (1–4 and 1–3,75, respectively, Fig. 1A,B). Additional distinct histopathological features of atherosclerotic plaque formation in rabbits were evaluated as well: cholesterol clefts were the most prominent finding and detected in 33.3% of all sections. Similarly, 29.2% showed neovascularization while microcalcification and hemorrhage were less frequent (16.7% and 12.5%, respectively; Fig. 1B). Peri-strut inflammatory reactions were mild to moderate, with a median score of 1 (1–2). Assessment of neointima formation by OCT confirmed histological results (Fig. 1). Neointimal area following BMS implantation was moderate, with a median area of 1.6 mm^2^ (1.5–2.2, Fig. 1C).

### Assessment of endothelial integrity *in-vivo*

**Figure 2:**
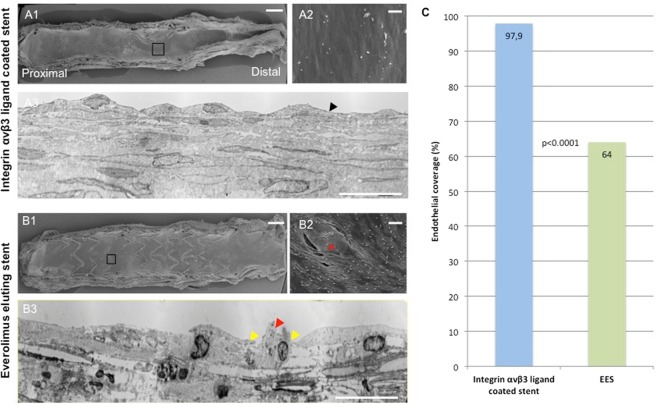
Comparison of integrin αvβ3 ligand coated stent (A1–A3) and EES (B1–B3) 12 weeks after implantation in a hypercholesterolemic rabbit model with quantification of endothelial coverage (**C**). Scanning electron microscopy (SEM) of an integrin αvβ3 ligand coated stent half (A1) and an Everolimus eluting stent (EES) half (B1) shows improved strut-coverage as compared to EES. High-magnification SEM images (A2 and B2) confirm a continuous monolayer of endothelial cells above integrin αvβ3 ligand coated stent struts whereas EES-struts seem to be covered by loosely arranged endothelial cells in the presence of scattered inflammatory cells and platelets (red asterisks = stent strut). Transmission electron microscopy (TEM) demonstrates a continuous endothelial monolayer with abundant intercellular junctions (arrowheads) in an integrin αvβ3 ligand coated stent (A3) while impaired endothelial monolayer integrity is observed in EES (B3, yellow arrowheads mark endothelial cells in the absence of intercellular junction, red arrowhead indicates incidental finding of a transmigrating monocyte). Scale bar: A1/B1 = 1 mm. A2/A3 = 25 µm. A3/B3 = 100 µm.

Mean cholesterol levels were 37.2 ± 7.3 mg/dl (baseline), 680.2 ± 150.3 mg/dl (denudation), 1872.3 ± 295.3 mg/dl (diet switch), 1110.0 ± 544.8 mg/dl (stenting) and 656.0 ± 198.8 mg/dl (termination). Evaluation of stented iliac arteries by confocal microscopy revealed substantial heterogeneity in vascular healing among commercially available EES and integrin αvβ3 ligand coated stents, where overall CD31 expression was significantly greater in integrin αvβ3 ligand coated stents as compared to EES (311.5 mm^2^ vs. 65.7 mm^2^, p < 0.05). Scanning electron microscopy confirmed the greater overall endothelial coverage of stent struts in integrin αvβ3 ligand coated stents compared to EES (97.9% vs. 64.0% covered stent struts, p < 0.0001) (Fig. 2A1,B1). High magnifications of representative SEM images confirmed the gaps in endothelial junctions in EES relative to integrin αvβ3 ligand stents (Fig. 2A2,B2), which was further confirmed by TEM (Fig. 2A3,B3).

**Figure 3:**
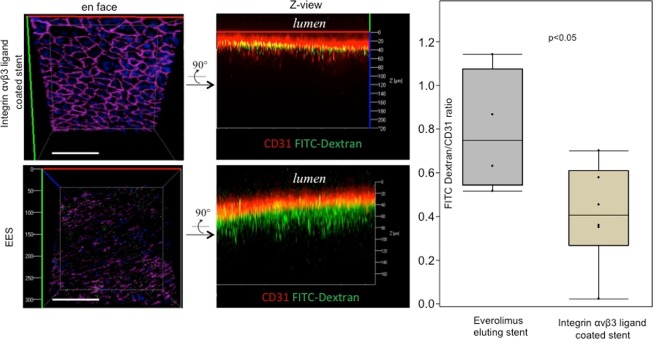
Left: Selective confocal microscopy images of an integrin αvβ3 ligand coated stent (top) and EES (bottom) 12 weeks after implantation in a hypercholesterolemic rabbit model. En face images (left) show strong CD31 staining of endothelial cells (cell shape, red channel, pink pseudocolor) in the integrin αvβ3 ligand coated stent and decreased CD31 staining in EES. FITC-dextran accumulation (green channel) between endothelial cells (red channel) is increased in EES as compared to integrin αvβ3 ligand coated stents (p < 0.05). n = 5 each and expressed as means with standard deviation calculated by ANOVA. Scale bar = 1 mm.

Co-registration of en face confocal microscopy and SEM images revealed a predominance of FITC-dextran deposition in arterial segments where CD31-positive endothelial cells were absent. The ratio of FITC-dextran/CD31 positive area*intensity was significantly greater in EES as compared to integrin αvβ3 ligand coated stents (0.73 vs. 0.40, p < 0.05) (Fig. 3). More importantly, Z-stack tile imaging provided evidence that most of FITC-dextran (green channel, Fig. 3) in EES was located underneath the endothelial monolayer (red channel, Fig. 3), which was not the case with integrin αvβ3 ligand coated stents, where the signal of endothelial cells was almost superimposed with that of FITC-dextran confirming the integrity of endothelial monolayer.

### Assessment of endothelial integrity *in-vitro*

**Figure 4:**
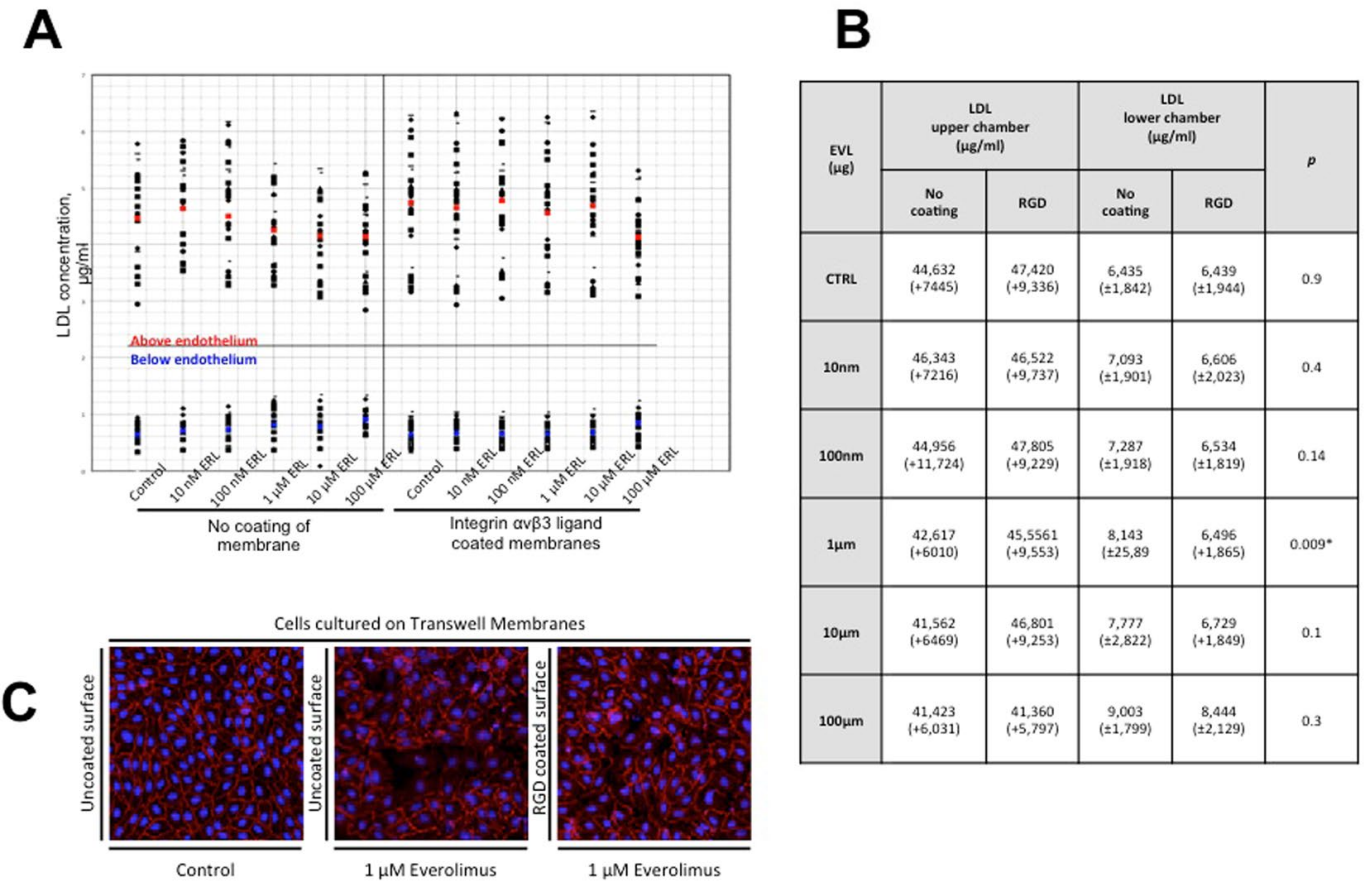
(**A,B)** AcLDL-concentrations in an *in vitro* permeability assay (transwell model) above and below endothelium which was cultured on ± integrin αvβ3 ligand coated semipermeable membranes and treated with everolimus in different concentrations (see B) for 24 h. Everolimus treatment causes a dose-dependent decrease of AcLDL in the upper compartment of the semipermeable membrane and an increase of AcLDL in the lower compartment (mean LDL concentration above endothelium marked in red and below endothelium in blue; n = 15). (**C**) Endothelial cells cultured on transwell membranes exposed to different concentrations of everolimus. The control group (uncoated surface) shows a confluent monolayer with intense VE-Cadherin staining (left). Incubation with everolimus at 1 µM for 24 h resulted in incomplete endothelial integrity on uncoated surfaces (centre image). Endothelial cells cultured on integrin αvβ3 ligand coated surfaces (right image) show preserved VE-Cadherin expression and less intercellular gaps (For results on quantification, please see Supplemental Fig. 3). Scale bar = 100 µm.

To subsequently verify that leaky endothelial junctions result in increased lipid uptake and foam cell transformation of monocytes, cell culture experiments were performed (for a schematic illustration of the transwell assay, please see Supplemental Fig. 2): confocal microscopy revealed a dose-dependent effect of everolimus on both HUVECs and HCAECs since organization of actin cytoskeleton (green phalloidin staining) as well as formation of adhesive cell-cell contacts (red VE-cadherin staining) were structurally modified and impaired. There was an inverse relationship of everolimus concentration and VE-cadherin expression, where highest concentrations of everolimus (100 µM) resulted in obvious gaps in the endothelial monolayer, which was observed on uncoated and RGD-peptide coated surfaces (Fig. 4C**)**. Cell quantification revealed a decrease in the number of cells with increasing doses of everolimus and higher amount of cells on RGD-peptide coated surfaces compared to uncoated surfaces (265 vs. 155 cells for 1 nm Everolimus and 140 vs. 38 cells for 10 µm Everolimus, Supplemental Fig. 3).

**Figure 5:**
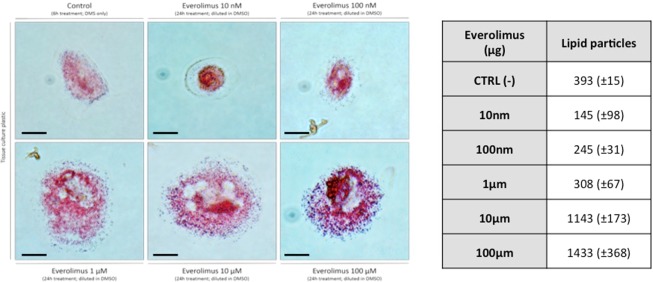
Brightfield images of foamy monocytes in the presence of AcLDL (24 h incubation on tissue culture plastic, n = 3) and results of automated quantification of lipid particles. Monocytes were co-cultured with endothelial cells after exposure to everolimus. Increasing concentrations of everolimus and fixed concentration of AcLDL result in dose-dependent transformation of monocytes into foam cells. (Foam cells stained with Oil-red-O, greater lipid accumulation in monocytes co-cultured with endothelial cells under high concentrations of everolimus). Scale bar = 20 µm.

Everolimus treatment of endothelial cells grown on uncoated and RGD-peptide coated membranes caused an increase in AcLDL-permeability. However, measurement of AcLDL concentrations above and beneath the endothelial monolayer showed a substantially decreased gradient when endothelial cells were grown on uncoated membranes (greater passage of AcLDL), while endothelial cells grown on RGD-peptide coated membranes not only showed more consistent confluence but also an increased gradient of AcLDL (decreased passage of AcLDL, Fig. 4A,B). This effect was seen on both, HUVECs and HCAECs. Co-cultures of monocytes and endothelial cells incubated with media containing increasing concentrations of everolimus and a fixed concentration of AcLDL, showed a dose-dependent transformation of macrophages into foam cells. Oil-red-O staining showed greater lipid accumulation in monocytes co-cultured with endothelial cells under high concentrations of everolimus as compared to those incubated with lower everolimus concentrations, which was further confirmed by automated quantification of lipid particles (145 vs. 245 vs. 308 vs. 1143 vs. 1433 lipid particles for 10 nm/100 nm/1 µm/10 µm/100 µm of everolimus, Fig. 5). This effect was seen in HUVECs and HCAECs.

## Discussion

In the current study we aimed to investigate whether acceleration of re-endothelialization via application of integrin αvβ3 ligand coating resulted in increased endothelial integrity and improved barrier function of the endothelial monolayer relative to conventional DES. With regards to this objective, the following salient findings can be summarized:

(i) Pro-healing integrin αvβ3 ligand coated stents resulted in augmented endothelial integrity as compared to commercially available EES and reduced deposition of FITC-dextran as a marker of endothelial permeability
(ii) Adjunct cell culture experiments confirmed the permeability of endothelial cells for AcLDL particles in the presence of increasing everolimus concentrations, which could partly be counterbalanced by the cell-adhesive properties of integrin αvβ3 ligand coating.
(iii) Exposure of macrophages co-cultures with endothelial cells incubated with increasing concentrations of everolimus resulted in dose-dependent foam cell transformation.

Neoatherosclerosis has recently been introduced as terminology to describe the manifestation of atherosclerosis within the nascent neointimal tissue in the aftermath of stent implantation. One of the key findings of first autopsy studies of patients dying of cardiac and non-cardiac death was the observation of a significant left shift in the cumulative frequency curve of neoatherosclerosis onset plotted against time in patients receiving first-generation drug-eluting stents (DES)^4^. This means that neoatherosclerosis was not only significantly more frequent in DES-treated patients but also occurred at shorter time intervals after stenting relative to matched BMS groups^9^. Neoatherosclerosis is associated with late-stent failure such as in-stent restenosis or stent-thrombosis^9,14,15^ Recently, a large registry investigating stent-thombosis by OCT found that very-late stent thrombosis (VLST) is caused by neoatherosclerosis in approximately 31% of cases, representing the predominant causative factor^14^.

This finding is alarming considering the large number of DES implanted on a global scale and means that a significant number of patients may be at risk for subsequent future events arising from stented coronary arteries.

Short on pathophysiological understanding of this novel disease manifestation, preclinical studies represent an important means to investigate the pathophysiology and key etiological factors facilitating neoatherosclerosis formation.

### Establishment of animal model

In this work we were able to reproduce early features of neoatherosclerosis by means of neointimal foam cell formation in a hypercholesterolemic animal model. Our model however (similar to many established atherosclerotic animal models) depends on supra-physiological cholesterol levels induced by dietary uptake and repeat endothelial denudation by balloon injury to mimic human atherosclerotic conditions. In contrast, atherosclerosis in humans often takes decades to develop and depends on additional important cofactors that cannot be reproduced in current animal models. In our study, neointimal foam cell formation was observed 13 weeks following study initiation, which represents a vastly accelerated course of neoatherosclerosis formation known from human pathology studies^9^. However, our model is also limited in the duration of cholesterol feeding owing to diet-induced liver failure and premature drop out of animals. Therefore, our current animal model is not able to mirror late stages of neoatherosclerosis, which are necessary to develop in-stent restenosis in man. Furthermore, the aim of the current study was not to quantitatively compare neoatherosclerosis formation among stent types but rather to provide insights into their differential endothelial healing and barrier function, which represent important preconditions of neoatherosclerosis formation.

### Defective endothelial barrier function

The integrity of the vascular endothelium is warranted by complex interactions of junctional proteins, which play a pivotal role in its permeability and vascular haemostasis. It is known that low concentrations of integrin αvβ3 ligands stimulate endothelial activation and stabilize endothelial cell barrier function (“vascular stabilisation”)^13,16^.

Whereas gap junctions are involved in intercellular exchange of smaller molecules, larger particles such as LDL are predominantly transported transcellular by the use of caveolae under physiologic conditions. During stent implantation, the vascular endothelium gets largely disrupted and regenerates over a variable time frame ranging from several weeks to months or even years in the presence of anti-proliferative drugs^6^. Delayed re-endothelialization has been described as a hallmark of increased thrombotic risk even late after DES implantation^10^, where the absence of junctional adhesive proteins has been shown to parallel decreased expression of anti-coagulatory markers in preclinical studies. The transmembrane protein platelet/endothelial cell adhesion molecule 1 (PECAM-1, CD31) is constitutively expressed along the intercellular junctions of endothelial cells, where it was shown to inhibit platelet aggregation in genetically engineered mice^17,18^. In the current study, we could show that disrupted integrity of the endothelial monolayer as exemplified by the absence of CD31 expression is giving rise to trans-endothelial permeability of dextran molecules in the range of 250–500 kDa, which resembles the size of LDL particles. In adjunct cell culture studies, we demonstrated foam cell transformation of human macrophages in the presence of high everolimus concentrations, where drug-induced endothelial toxicity was likely the key phenomenon explaining increased permeability of LDL particles. Whether the occasional absence of endothelial cells after apoptotic cell death or their unstable intercellular junctions among viable cells were paramount in this process remains to be determined in future dedicated studies.

### The relevance of stent coating

Endothelial function is largely supported by integrins, a family of heterodimeric transmembrane receptors^7,19^. One of the abundantly expressed receptor subtypes on vascular endothelial cells is the integrin αvβ3 that stabilizes endothelial cells under stress^13,16,20^ and facilitates anchorage of endothelial cells to the extracellular matrix^21^. This interaction is mediated by the RGD integrin-binding motif (Arg-Gly-Asp) which is an integral component of several extracellular matrix compounds and is leveraged to promote cellular adhesion using integrin αvβ3 ligand coating of stent surfaces^7^. In the current study a cyclic αvβ3 ligand was applied which is highly specific for integrin αvβ3; it has been reported previously that cyclic RGD peptides specific for integrin αvβ3 foster endothelialization of stent surfaces^7,19,22^. Consequently, integrin αvβ3 represents a passive pro-healing coating technology to facilitate vascular healing following stent implantation and was not only shown to increase endothelial cell attachment but rather improve its integrity. Whether improved endothelial integrity achieved by integrin αvβ3 coating results in decreased neoatherosclerosis formation needs to be determined in dedicated preclinical studies. Our findings are supported by other studies, which have shown a beneficial effect of integrin αvβ3 in sepsis through vascular stabilization, eventually preventing endothelial leakage^23–25^.

*In-vitro* studies focusing on immunosuppressive drug effect on endothelial barrier function showed a protein kinase C mediated destabilization of the p120-VE cadherin interaction causing internalization of VE-cadherin and, consequently, impaired endothelial integrity^26^. These findings strengthen the hypothesis that DES are especially susceptible to neoatherosclerosis because of impaired endothelial barrier function. While the precise molecular mechanisms underlying this pathophysiology remain to be determined, the current study suggests that a prolonged delay of endothelial integrity may play a pivotal role for premature onset of neoatherosclerosis formation in current generation DES. Inflammation is another important component of atherosclerosis and was shown to be of relevance during neoatherosclerosis formation in the currently applied animal model. While the differential response to hypercholesterolemic feeding and procedural interventions likely determined the magnitude of neointimal foam cell formation in the current study, the exact impact of stent-induced inflammatory reactions on foam cell transformation of monocytes could not be assessed in the current study and may have played an important role.

While clinical translation of pro-healing stents has been shown to be feasible and safe (such as the HEALING-FIM study investigating endothelial progenitor cell (EPC) capturing stents^27^), targeting endothelial healing with novel stent-based approaches has however not been proven to be clinically effective in terms of cardiovascular event reduction or decrease in revascularization procedures. In the most recent HARMONEE trial, the dual therapy COMBO DES using luminal anti-CD34 antibody coating to capture endothelial progenitor cells has proven clinical equipoise with regards to classical patient and device-oriented endpoints, however superiority with regards to these endpoints could not be achieved (30). Healthy strut coverage defined as strut coverage with thickness of greater than 40 μm above stent struts was shown to be superior in COMBO vs. Xience DES. The hypothetical advantage of using DES, which fosters vascular healing, may only be proven at long-term follow up since progression of atherosclerosis within stented vascular segments takes years to manifest clinically (31). We also agree that stent failure is likely multi-factorial, where formation of neoatherosclerosis is only one factor among others to contribute to this dire phenomenon. Improvement of stent technology alone may not be sufficient to overcome the sustained increase in cardiovascular events over time observed with implantation of DES. Optimized preventive strategies focusing on individualized patient care and novel pharmacological approaches may be used in synergy with novel stent designs to tackle this significant clinical problem.

## Supplementary information

Supplementary Information.

Supplementary Figure 1.

Supplementary Figure 2.

Supplementary Figure 3.
