# Supplementary material for: Assessment of a pro-healing stent in an animal model of early neoatherosclerosis
Source: Sci Rep. 2020 May 19;10:8227. doi: 10.1038/s41598-020-64940-2 (PMC7237429; doi:10.1038/s41598-020-64940-2)
Supplement: Supplementary file 1 — Supplementary Information. [file 41598_2020_64940_MOESM1_ESM.docx]

**Assessment of a pro-healing stent in an animal model of early neoatherosclerosis**

**- SUPPLEMENT -**

Philipp Nicol^a^, MD; Christoph Lutter^g^, MD; Anna Bulin^a^, DVM; Maria Isabel Castellanos^a,c^, PhD; Tobias Lenz^a^, MD; Petra Hoppmann^b^, MD; Anna Lena Lahmann^a^, MD; Roisin Colleran^a^, MD; Kristina Euller^a^, MD; Kristin Steigerwald, DVM^a^; Stefanie Neubauer^d^, PhD; Florian Rechenmacher^d^, PhD; Beatrice Stefanie Ludwig^h^; Michael Weinmüller^d^, Dipl.-Chem.; Garry Kerch^e^, PhD; Liang Guo^f^, PhD; Qi Cheng^f^, MD; Eduardo Acampado^f^, DVM; Tobias Koppara^b^, MD; Horst Kessler^d^, PhD; Michael Joner^a,c^, MD

a) Klinik für Herz- und Kreislauferkrankungen, Deutsches Herzzentrum München, Technische Universität München, Germany 
b) Klinik und Poliklinik für Innere Medizin I, Klinikum rechts der Isar, Technische Universität München
c) DZHK (German Centre for Cardiovascular Research), partner site Munich Heart Alliance, Munich, Germany
d) Institute for Advanced Study, Department of Chemistry and Center for Integrated Protein Science, Technische Universität München, Germany
e) Riga Technical University, Riga, Latvia
f) CVPath Institute Inc., Maryland, USA.
g) Department of Orthopedics, University Medical Center, Rostock, Germany
h) Klink für Nuklearmedizin, Klinikum rechts der Isar, Technische Universität München

## Supplementary methods

**Optical Coherence Tomography**

Frequency domain optical coherence tomography (OCT) was performed in all animals in both the stented iliac arteries prior to termination. The stented artery was engaged with a guiding catheter and the OCT catheter (2.7-F, St. Jude Medical, USA) was delivered to the stented segment over a 0.014 inch guidewire following angiography. A pullback was then performed from the distal to the proximal arterial segments to include the stented portion of the artery at a pullback speed of 15mm/s (120 frames/second) after confirmation of correct catheter position. For improved image quality, vessels were simultaneously flushed with contrast at low pressure. OCT images were adequately recorded and stored for further investigation and co-registration. Computer-assisted software for offline analysis (Medis medical imaging systems, Leiden, Netherlands) was used for OCT image analysis. Quantitative measurements included the proximal and distal vessel reference area. Within the stented segment, five frame measurements were performed and included vessel lumen and stent area. Percentage area stenosis was calculated as: (1 - lumen area/ stent area)*100. Neointimal thickness was measured from the hyperreflective luminal border of stent struts to the endoluminal surface applying the edge-to-edge principle.

**Coating of stents with αvβ3 integrin ligand**

ProKinetic bare metal stents (3.0x15mm) were coated with the chitosan-polylactide copolymer (Kytozyme, NH_2_/C= 5.0 %) using layer-by-layer deposition technology. To increase the density of reactive (NH_2_)-groups for coupling of cyclic RGD peptides, plasma treatment was performed in a customer-specific version of the V55G low-pressure plasma processor (PINK GmbH Vakuumtechnik, Wertheim-Reinhardshof, Germany). The microwave plasma source was located on the top of the chamber. It used a parabolic microwave field reflector to generate large-area planar microwave plasma below the coupling window. The base pressure of the reactor was 0.1 mbar (1). The plasma was operated at a microwave power of 500 W. The substrate position was 5 cm below the coupling window for the microwaves. A gas flow of 40 ml NH_3_ per minute was applied at a constant pressure of 0.2 mbar. The treatment time was 30 s. To guarantee a homogeneous treatment of the stents, a special sample holder was designed and manufactured where the stents stand vertically inside the vessel. Following plasma treatment, stents were immediately immersion-coated with the cyclic integrin αvβ3 RGD peptide, *c*(RGDfK)‑Ahx‑Ahx‑1‑(4‑isothiocyanatophenyl)thioureidyl (synthesis in detail see below),) at a concentration of 10 μm for 24 hours, followed by generous rinsing with sterile PBS solution. Following crimping of stents, sterilization was performed using ethylene oxide sterilization. Stable coating of stents by the cyclic RGD peptide relative to uncoated stents was confirmed by in vitro cell adhesion assays (data not shown).

**Synthesis of cyclic RGD peptides**

*General Procedure*

**GP1.** *Loading of TCP-resin.* Peptide synthesis was carried out using TCP‑resin (~80% maximum loading capacity) following standard Fmoc-strategy. Fmoc‑Xaa‑OH (1.2 eq.) were attached to the TCP-resin with *N*,*N‑*diisopropylethylamine (DIEA; 2.5 eq.) in anhydrous DCM (8 mL/g resin) at room temperature for 1 h. Afterwards the resin is capped by addition of a solution of MeOH (0.2 mL/g resin) and DIEA, (5:1; v:v), for 15 min. The resin was filtered and washed with DCM (5x), NMP (3x), NMP/MeOH 1:1 (1x) and with MeOH (3x). The loading capacity was determined by gravimetric method after drying the resin under vacuum.

**GP2.** *On-resin Fmoc Deprotection.* The Fmoc peptidyl-resin was treated with 20% piperidine in NMP (v/v) for 15 min and a second time for 10 min. The resin was washed following with NMP (5x).

**GP3.** *Standard Amino Acid Coupling.* A solution of Fmoc-Xaa-OH (2 eq.), *O*-(7-azabenzotriazole-1yl)-*N*,*N*,*N’*,*N’*-tetramethyluronium-hexafluorophosphate (HATU) (2 eq.), 1-hydroxy-7-azabenzotriazole (HOAt; 2 eq.), and DIEA (5 eq.) in NMP (1 mL/g resin) was added to the free amino peptidyl-resin and shaken for 60 min at room temperature and washed with NMP (5x).

**GP4.** *Cleavage of Linear Peptides from Resin.* For complete cleavage from the resin the peptides, which were washed with DCM (3x), were treated three times with a 20% hexafluoroisopropanol (HFIP) solution in DCM at room temperature for 10 min, the resin was washed afterwards with DCM (3x) and the solvent of the combined solutions is evaporated under reduced pressure.

**GP5.** *Reductive Deprotection.* The orthogonal deprotection of the benzyl-group via hydrogenolysis was performed using a palladium catalyst on activated carbon (5% Pd/C, 15 mg/mmol) and hydrogen atmosphere (1 atm H_2_) at room temperature. The completion of the deprotection was monitored by TLC or HPLC-MS, respectively, the catalyst was removed over *Celite* and the solvent was removed under pressure.

**GP6.** *Coupling with HATU in solution.* The unprotected amine is dissolved in DMF (~0.1 m) and the corresponding acid (1.2 eq.), HATU (1.2 eq.) and DIEA (5 eq.) are added. The reaction solution is mixed at room temperature for 16 h, following the solvent is removed under reduced pressure, the residue is dissolved in ethyl acetate and is extracted with saturated NH_4_Cl and NaHCO_3_ solution. Afterwards it is washed with saturated NaCl solution (*brine*), dried with Na_2_SO_4_ and the solvent is reduced under reduced pressure. Final products are purified via semi-preparative HPLC and are lyophilized. A TFA salt as a final product is received.

**GP7.** *Removal of Acid Labile Side Chain Protecting Group.* Cyclized peptides were stirred in a solution of TFA, water and TIPS (95/2.5/2.5, v/v/v) at room temperature for 1 h or until no more protected peptide could be observed by HPLC-MS. The solvent is removed under reduced pressure and is co‑evaporated with toluene (3x).

**Spacer-Linker unit: HO-Ahx-Ahx-1-(4-isothiocyanatophenyl)thioureidyl**

The spacer-linker unit is synthesized following standard Fmoc strategy on solid resin: 1.00 g TCP-Harz (~80% maximum loading capacity) is loaded with Fmoc‑Ahx‑OH (6-(Fmoc-amino)hexanoic acid) according to **GP1**. Afterwards the protection group Fmoc is deprotected according to **GP2** and Fmoc‑Ahx‑OH is coupled according to **GP3**. After the deprotection of Fmoc (**GP2**) and the coupling with 1,4-phenylene diisothiocyanate (5 eq.) and DIEA (3 eq.) in DCM for 1 h at room temperature, the spacer-linker unit is cleaved with HFIP from the resin (**GP4**) and is co-evaporated with toluene (2). The yield is 93.0 mg (0.2 mmol, 25% referred to the resin loading).

**HPLC** (10-90%, 15 min): t_R_ = 7.34 min, **MS** (ESI): m/z = 437.1 [m+H]^+^.

***Cyclic integrin αvβ3 peptide: c*(R(Pbf)GD(*t*Bu)fK)**

The protected cyclic pentapeptide, *c*(R(Pbf)GD(*t*Bu)fK), is synthesized according to the literature. [*R. Haubner, R. Gratias, B. Diefenbach, S. L. Goodman, A. Jonczyk, H. Kessler, J. Am. Chem. Soc.****1996****, 118, 7461‑7472.*] However, the side chain protection groups, Pbf (2,2,4,6,7-pentamethyldihydrobenzofuran‑5‑sulfonyl) and *t*Bu (*tert.*-butyl), are in this synthesis procedure not deprotected except the Cbz (carboxybenzyl) group of Lys which is deprotected according to **GP5**. The yield is 0.40 g (0.43 mmol, 54% referred to the resin loading). The received raw product is used without further purification in the next synthesis steps.

**HPLC** (10-90%, 30 min): t_R_ = 19.46 min, **MS** (ESI): m/z = 912.4 [m+H]^+^, 856.4 [m-*t*Bu+H]^+^.

**Cyclopeptide with isothiocyanate functionalization for facilitated anchorage to amino groups:**

***c*(RGDfK)‑Ahx‑Ahx‑1‑(4‑isothiocyanatophenyl)thioureidyl**

The cyclopeptide, *c*(R(Pbf)GD(*t*Bu)fK), (41.8 mg, 45.8 µmol, 1 eq.) and the linker unit, HO-Ahx-Ahx-1-(4-isothiocyanatophenyl)thioureidyl, (20.0 mg, 45.8 µmol, 1 eq.) are coupled according to **GP6** with additional HOAt (1.2 eq.). Following, the compound is deprotected according to **GP7** and cleaned via semi-preparative HPLC. The yield is 4.20 mg (4.11 µmol, 9%).

**HPLC** (10-60%, 30 min): t_R_ = 25.68 min, **MS** (ESI): m/z = 1022.5 [m+H]^+^.

**Confocal Microscopy**

The specimens were mounted “en face” on glass slides and representative images were acquired with a Zeiss LSM 700 laser confocal microscope equipped with ZEN imaging software (ZEN 2011 edition, Carl Zeiss Microscopy - Germany). Proximal segments adjacent to the stent served as positive control for immunostaining. Short segments of the aorta served as negative control in the absence of primary or secondary antibody. For imaging analysis purposes, confocal z-stack images with tile imaging were acquired at x10 magnification. Representative high power fluorescent images of the luminal surface from (proximal, middle, and distal) regions of the artery were also acquired by confocal microscopy using a x20 objective with z-stack acquisition to document the ultrastructural spatial distribution of CD31 and FITC-dextran. CD31 expression and FITC-dextran distribution were analyzed by ZEN image analysis tool (Zen 2012 - Carl Zeiss Microscopy, Germany) and reported as a ratio of FITC-dextran positive area (multiplied by its mean intensity) in the green channel relative to CD31 positive area (multiplied by its mean intensity) in the red channel. For in-vitro analysis of intercellular junctions, fluorescently labelled LDL particles and nuclei, Zen 2012 imaging software was used (Carl Zeiss Microscopy, Germany).

**Scanning Electron Microscopy**

Following confocal microscopy, samples were demounted from glass slides and generously rinsed in PBS, followed by 0.1M sodium phosphate buffer and post-fixed in 1% osmium tetroxide for approximately 30 minutes. The samples were then dehydrated in a graded series of ethanol, critical point dried, and sputter-coated with gold. The specimens were visualized using a Hitachi Model S3400N or S3600N scanning electron microscope. Low power photographs of x15 magnification were taken of the luminal surface to estimate the degree of neointimal incorporation of the implant. Regions of interest were photographed at incremental magnifications of x50, x200 and x600 magnification. Endothelial cells were identified as sheets of spindle or polygonal shaped monolayers in close apposition, a distinguishing feature in contrast to other cell types in en face preparations. Quantification of endothelial coverage was achieved with the help of a customized software algorithm (ImageJ 1.5, NIH, USA). Strut endothelialization was derived from the total area of endothelialization minus the area between stent struts.

## Supplementary figures

**Supplemental Figure 1**

Left: Animal study flow of study for establishment of neointimal foam cells in n=7 rabbits using BMS (n=14). Right: Proof-of-principle study using n=5 rabbits randomized to EES (n=5) or ICS (n=5).

**Supplemental Figure 2**

Schematic figure of the in vitro permeability assay (transwell model, n=3) (**A1**) Endothelial cells were cultured on ± integrin αvβ3 ligand coated semipermeable membranes and treated with everolimus in different concentrations. (**A2**) Trans-cellular passage of AcLDL is prevented in the presence of a continuous endothelial monolayer whereas impaired endothelial integrity leads to an increased AcLDL passage (**A3**). (**A4**) AcLDL presence in the cell culture media leads to a dose-dependent transformation of monocytes into foam cells.

**Supplemental Figure 3**

Automated quantification of cell density between RGD-coated and uncoated membranes with increasing doses of everolimus.

## References:

1. Meyer-Plath AA, Schröder K, Finke B, Ohl A. Current trends in biomaterial surface functionalization - Nitrogen-containing plasma assisted processes with enhanced selectivity. User Model User-adapt Interact. 2003;71(3):391–406.

2. Kalinina S, Gliemann H, López-García M, Petershans A, Auernheimer J, Schimmel T, Bruns M, Schambony A, Kessler H, Wedlich D. Isothiocyanate-functionalized RGD peptides for tailoring cell-adhesive surface patterns. Biomaterials. 2008;29(20):3004–13.
