# Supplementary figures and images for: Assessment of a pro-healing stent in an animal model of early neoatherosclerosis
Source: Sci Rep. 2020 May 19;10:8227. doi: 10.1038/s41598-020-64940-2 (PMC7237429; doi:10.1038/s41598-020-64940-2)

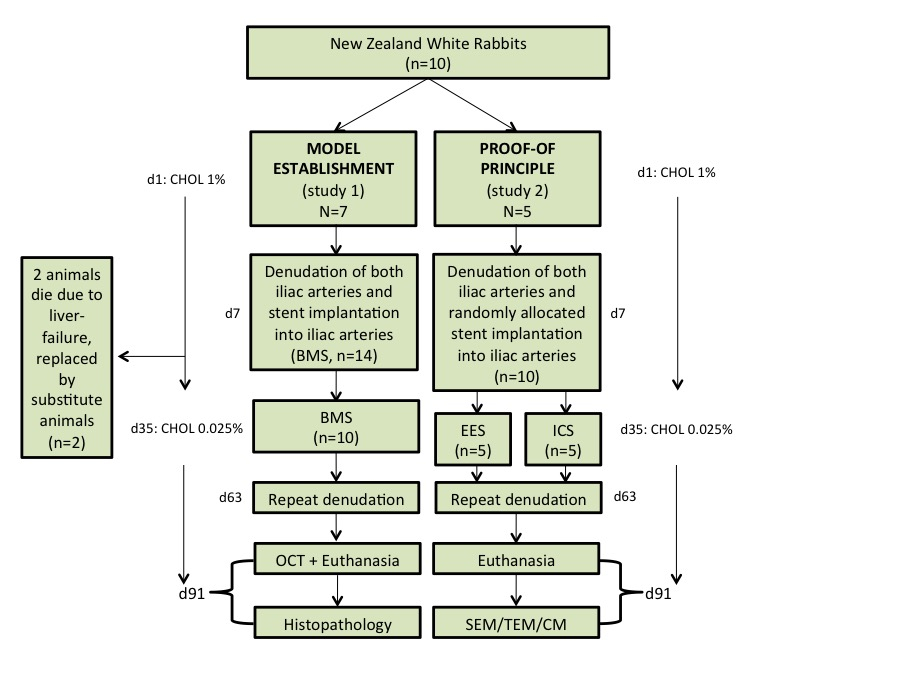

Supplement: Supplementary file 2 — Supplementary Figure 1. [file 41598_2020_64940_MOESM2_ESM.jpg]

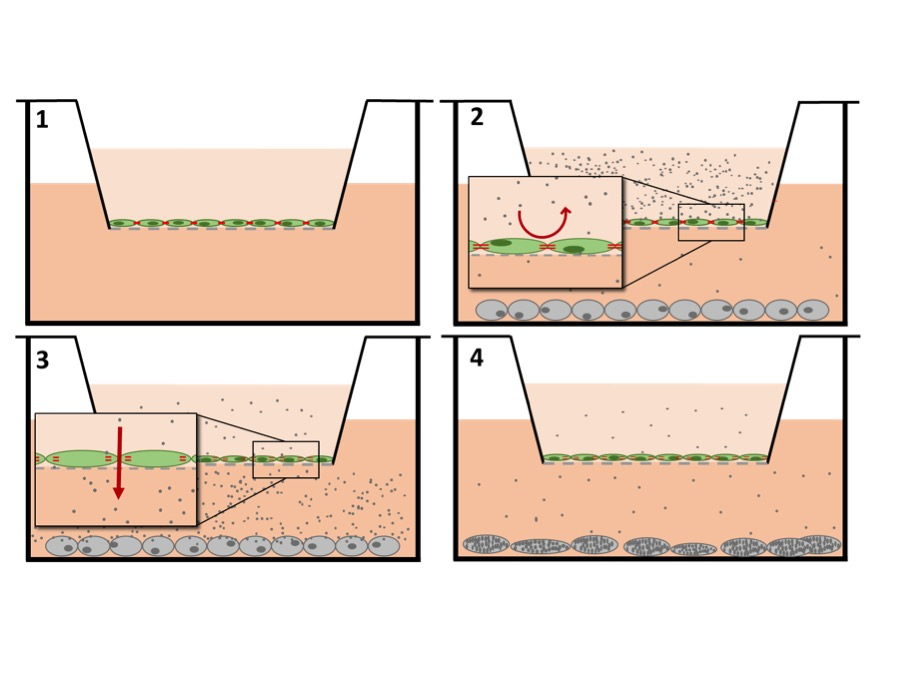

Supplement: Supplementary file 3 — Supplementary Figure 2. [file 41598_2020_64940_MOESM3_ESM.jpg]

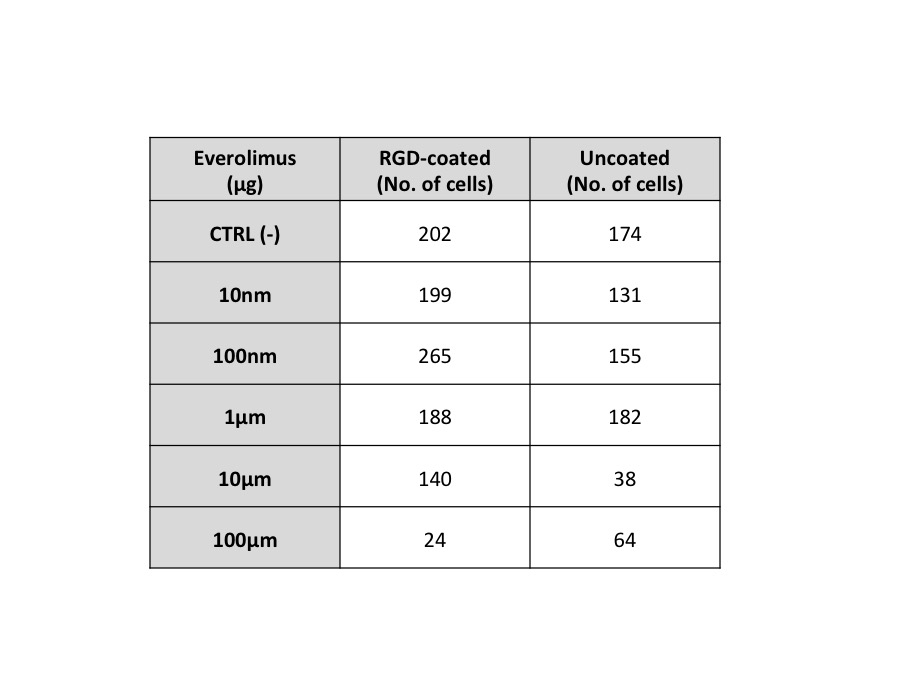

Supplement: Supplementary file 4 — Supplementary Figure 3. [file 41598_2020_64940_MOESM4_ESM.jpg]
